# Inhomogeneous Response of Articular Cartilage: A Three-Dimensional Multiphasic Heterogeneous Study

**DOI:** 10.1371/journal.pone.0157967

**Published:** 2016-06-21

**Authors:** Sara Manzano, Monica Armengol, Andrew J. Price, Philippa A. Hulley, Harinderjit S. Gill, Manuel Doblaré, Mohamed Hamdy Doweidar

**Affiliations:** 1 Mechanical Engineering Department, School of Engineering and Architecture (EINA), University of Zaragoza, Spain; 2 Aragón Institute of Engineering Research (I3A), University of Zaragoza, Spain; 3 Biomedical Research Networking Center in Bioengineering, Biomaterials and Nanomedicine (CIBER-BBN), Spain; 4 Department of Engineering Science, University of Oxford, Oxford, United Kingdom; 5 Nuffield Department of Orthopaedics, Rheumatology and Musculoskeletal Sciences, University of Oxford, Nuffield Orthopaedic Centre, Oxford, United Kingdom; 6 Department of Mechanical Engineering, University of Bath, Bath, United Kingdom; University of California, UNITED STATES

## Abstract

Articular cartilage exhibits complex mechano-electrochemical behaviour due to its anisotropy, inhomogeneity and material non-linearity. In this work, the thickness and radial dependence of cartilage properties are incorporated into a 3D mechano-electrochemical model to explore the relevance of heterogeneity in the behaviour of the tissue. The model considers four essential phenomena: (i) osmotic pressure, (ii) convective and diffusive processes, (iii) chemical expansion and (iv) three-dimensional through-the-thickness heterogeneity of the tissue. The need to consider heterogeneity in computational simulations of cartilage behaviour and in manufacturing biomaterials mimicking this tissue is discussed. To this end, healthy tibial plateaus from pigs were mechanically and biochemically tested *in-vitro*. Heterogeneous properties were included in the mechano-electrochemical computational model to simulate tissue swelling. The simulation results demonstrated that swelling of the heterogeneous samples was significantly lower than swelling under homogeneous and isotropic conditions. Furthermore, there was a significant reduction in the flux of water and ions in the former samples. In conclusion, the computational model presented here can be considered as a valuable tool for predicting how the variation of cartilage properties affects its behaviour, opening up possibilities for exploring the requirements of cartilage-mimicking biomaterials for tissue engineering. Besides, the model also allows the establishment of behavioural patterns of swelling and of water and ion fluxes in articular cartilage.

## Introduction

Articular cartilage is a highly heterogeneous, anisotropic and multiphasic material mainly composed of a solid phase (collagen fibrils and glycosaminoglycans (GAGs)), a fluid phase (interstitial liquid) and an ionic phase (Na^+^, Cl^-^, Ca^+^ and K^+^ among others) [1–3]. This composition and the associated structure determine the heterogeneous mechano-electrochemical properties and the inhomogeneous behaviour of the tissue [4, 5]. The main components involved in the swelling process of this tissue are a dense collagen network with attached GAGs, ions and the interstitial liquid that transports them, and oxygen and nutrients for tissue maintenance [6, 7]. The difference in ion concentration between the extra-cellular matrix and the interstitial liquid induces an osmotic pressure variation, also known as Donnan osmotic pressure. From the biological point of view, swelling is an essential process for the exchange of essential nutrients, oxygen, carbon dioxide and waste products in articular cartilage [8]. It has been reported that swelling is a good indicator of tissue quality [9–11]. When cartilage is degraded (for instance in advanced grades of osteoarthritis, long-term immobilized patients and severe cartilage degenerative diseases), collagen fibers and proteoglycans (PGs) lose their ability to retain water molecules. As a result, the physiological hydration of the cartilage increases [12–17]. In contrast, in the case of short-term immobilized patients and early osteoarthritis, the matrix becomes stiffer, exerting higher resistance to fluid passage [12, 13, 18, 19]. Despite intensive research carried out on cartilage-like biomaterials, the success of new biomaterials in mimicking cartilage behaviour remains elusive. No effective constructs for chondrocyte growth have been developed and advances in cartilage-mimicking biomaterials are limited [20]. The main reason seems to be directly related to the lack of consensus on simple answers to basic questions such as: what is the role of the inhomogeneity of the tissue in swelling? How do variations in tissue properties affect ion fluxes? Does neglecting these variations, when designing cartilage-mimicking materials, partly account for their lack of success? Three-dimensional (3D) computational models can provide additional insight into how the variation in the biomaterial composition affect its behaviour when implanted in the human body. Numerical modelling allows this to be done in an accurate and time efficient manner [8, 21, 22]. In the last decade, the majority of works related to articular cartilage simulations where developed in 2D. However, in the last years, advances in this field toward more accurate 3D simulations have achieved, which leads to improvement in the studying of specific cartilage phenomena as swelling [23, 24], neglecting important phenomena which occur under more realistic 3D environmental conditions. Moreover, most currently reported models consider cartilage as a homogeneous material, so the spatial variation in its properties is neglected [18]. More models that are recently incorporate transverse anisotropy, considering different cartilage layers, while neglecting the existing radial variations [6, 7]. In this paper, we present a novel 3D mechano-electrochemical model (based on our previous model [12, 13]) which aims to address these important questions and to assist in the analysis of the effects of articular cartilage anisotropy and inhomogeneity on tissue behaviour. In this respect, we present a novel 3D model that includes all the essential features of the articular cartilage, namely: (i) Donnan osmotic pressure, (ii) convective and diffusive processes, (iii) variations of chemical expansion due to the heterogeneous distribution of PGs and (iv) three-dimension through-the-thickness heterogeneity of the tissue. To achieve these goals, several experiments were conducted to measure variations in the properties along the cartilage surface and through the thickness. Specifically, indentation tests were carried out to obtain Young´s modulus (*E*_*s*_) and histological analysis of tissue samples was undertaken to qualitatively establish the GAG distribution in six tibial plateaus from porcine specimens. These properties were included in the model to analyse their influence on free swelling, on water and cation fluxes and ion distribution. A comparison between the obtained results and the results of the homogeneous behaviour were performed. They showed significant lower swelling in stiffer areas. Indeed, swelling was altered by the lower water and ion fluxes that appear in such specific areas of samples. Furthermore, the cation distribution in the heterogeneous configuration has more significant alterations compared to that obtained with homogeneous simulations. In the former case, the cation distribution is significantly reduced in magnitude.

## Material and Methods

Detailed experiments were conducted to measure *E*_*s*_ and the qualitative GAG distribution in the three-dimensional environment of the tissue for six porcine tibial plateaus obtained commercially from an abattoir (W.H.Alder in Cowley Road, Oxford). A Whole Articular Surface Indentation Machine (WASIM), specially designed to scan and indent articular surfaces, was used to measure the *E*_*s*_. Qualitative GAG distributions were obtained from histological assays of samples extracted from these tibial plateaus after the mechanical testing had been completed. Note that specific values for GAG concentration within the sample and the subsequent initial fixed charge density ($c_{0}^{F}$) are included in the computational model were estimated following the method of Lai et al. [25] as well as our own histological results. This means that qualitative GAG distribution and the subsequent $c_{0}^{F}$ distribution in cartilage zones are included in our model.

The WASIM was specifically designed to measure mechanical properties of intact specimens. Due to its five degrees of freedom, in-situ mechanical properties can be obtained over the tibial surface using indentation normal to the surface at any given point on the articular surface.

The WASIM consists of a stage that can translate in the X and Y directions and rotate in pitch. The sample holder located in the middle of the stage can rotate about its central longitudinal axis and thus provide yaw rotations. The indenter is mounted so that it can translate in the Z direction, but is fixed in the X and Y directions. Indentation tests were carried out using a hemispherical indenter (1.35 mm radius). The WASIM also incorporates a high resolution laser (LK-G32, Keyence, Higashi-Nakajima, Japan) for measuring the height of the specimen surface (i.e. measurement in the Z direction).

The high resolution laser combined with raster scanning along the X and Y axes provides a high-accuracy topographical map of the tibial surface. Using this topographical map, test points over the tibial plateau were selected and normal vectors of test points were calculated. Using these normal vectors, the WASIM rotation angles and translations were calculated to achieve normal indentation at the selected test points. *E*_*s*_ was obtained using the Field and Swann method and was adjusted by cartilage thickness measured post-hoc:

$$E\;=\;\frac{1-v^{2}}{2}\;\sqrt{\frac{\pi }{A_{c}}}\frac{dP}{dh}$$

Where *ν* is the Poisson’s modulus (set to 0.4), *P* is the force, *h* is the penetration and *dP*/*dh* corresponds to the unloading curve of the force-displacement curve. *A*_*c*_ is the area of compression given by *πa*^2^ and was calculated from Filed and Swain method [26, 27].

The solid phase heterogeneity is an important aspect to be included in accurate cartilage simulation. This was indirectly considered in this model through the heterogeneous *E*_*s*_ and $c_{0}^{F}$ distribution. Both parameters vary though the thickness and in radial direction due to the different collagen fibers orientation and distribution. In this sense, experimental measures reveal this collagen distribution providing different rigidity in both directions.

### Sample description

Experimental tests were performed on six porcine tibial plateaux. For this reason, pig legs were purchased from a commercial abbatoir (W.H.Alder in Cowley Road, Oxford) following routine humane slaughter. No approval process or legislation governs use of commercial butcher’s meat for experimental use post slaughter. The porcine specimens were males aged between 6 month and 1 year. Porcine specimens were selected due to their greater similarities with human tibial plateau in terms of size and shape compared with ovine or equine species [28]. The porcine knee joints were dislocated and dissected to obtain intact tibial plateau with subchondral bone and tibial shank.

### Cartilage sample preparation

Soft tissues, ligaments and menisci were removed from tibial plateau samples. They were then fixed onto the sample holder of the WASIM using bone cement (VersoCit-2 kit, Struers, UK). The cartilage was kept hydrated throughout the indentation process, by regularly applying saline solution (0.15 M) to the sample. After mechanical tests were performed, four millimetre diameter cartilage/subchrondral bone plugs, centred on previous indentation points, were extracted from the tibial plateau using an osteotome.

### Indentation test

**Fig 1:**
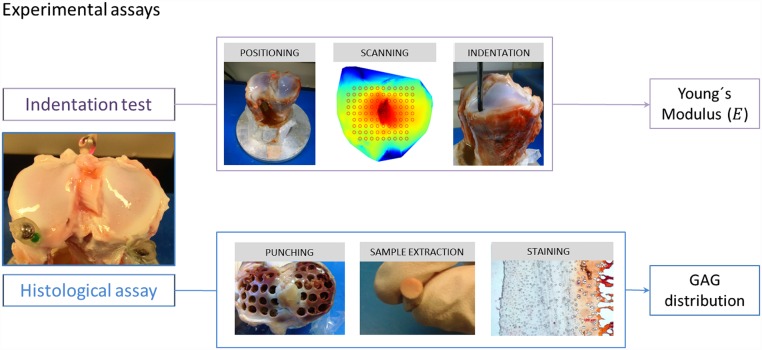
Intact porcine tibial plateau sample used for indentation and histological tests. The red circle indicates the area of interest where indentation tests were performed.

**Fig 2:**
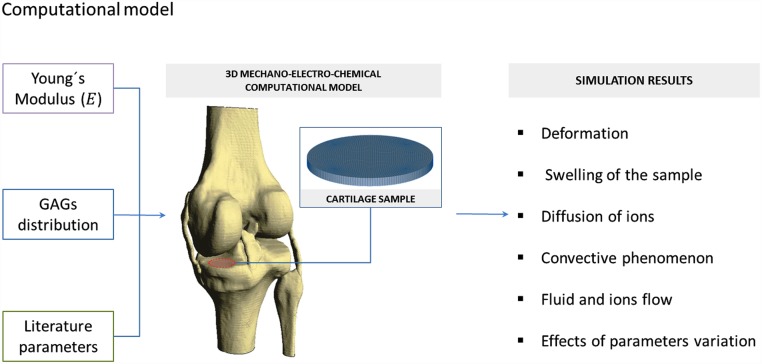
Axial view of the topographical map of the joint surface and the specific points selected for indentation.

Displacement controlled indentation was carried out at 16 points of measurement in the upper surface of the cartilage samples up to 10% maximum strain (Figs 1 and 2). Thickness of the sample was measured after mechanical tests were performed, therefore an adjustment due to articular cartilage thickness was carried out. Displacement controlled indentation was applied at a rate of 10 percent per second (pps). This rate allows the assumption cartilage behaviour as an elastic material at each test point. Note that in this experiment we consider that the majority of water content placed in the superficial zone of the samples is exuded at the very beginning of the process. Thus, the resulting measurement of mechanical properties refer to a superficial zone conformed by collapsed collagen fibrils, assuming almost null water content.

To estimate the mechanical properties in sample thickness (E_depth_ and ν_depth_), the following equations were used [28],

$$E_{depth}=E_{sup}\left(1+\alpha_{E}\frac{z}{h}\right),$$ (1)

$$\nu_{depth}=\nu_{sup}\left(1+\alpha_{\nu }\frac{z}{h}\right),$$ (2)

where *E*_*sup*_ and *ν*_*sup*_ are the Young´s modulus and Poisson ratio at the articular cartilage surface, respectively; *h* is the thickness of the sample; *z* denotes the depth of the layer considered and *α*_*E*_ and *α*_*ν*_ represent specific material constants (*α*_*E*_ ≥ 0; α_*ν*_ ≥ 0). For this simulation, *α*_*E*_ = 3.2 and *α*_*ν*_ = 2.8 [29]. Indentation tests were performed to measure top layer properties (*E*_*sup*_ and *ν*_*sup*_) Then, these superficial values were applied in Eqs (1) and (2) to estimate mechanical properties of the sample through the thickness. For the homogeneous simulation, *E* was obtained from a long time experimental indentation, which provides this parameter considering the sample as homogeneous material. In contrast, for heterogeneous simulations, this value was extracted for each discrete region of cartilage top layer and then mathematically extrapolate to get values in depth.

### Histological analysis

Samples were prepared for histological analysis to observe cross-sectional GAG distribution. The cartilage/bone plugs were immersed in 10% neutral buffered formalin and 4% EDTA for 72 hours to decalcify. Samples were then cryofrozen and sectioned into 5 *μ*m thick sections for staining with Safranin O. It has been shown that these techniques of fixation and decalcification has a small effect in GAG content [30, 31]. Finally, the samples were observed under 40x and 20x magnification using a light Olympus BX40F4 microscope. Histological images were recorded using an Olympus U-READDB 60X camera. Colour gradients were obtained indicating the degree of GAG concentration within the samples. Based on this obtained qualitative distribution, the specific values for the concentrations of the fixed charges attached to the GAGs were obtained from Lai et al. [25].

### Computational formulation of the Mechano-electrochemical model

The main model formulation is explained below, building further on our previous work [12, 13]. The model was based on the triphasic theory for soft, charged and hydrated tissues [21]. Four specific components were considered: negatively charged porous-elastic solid (s), fluid (f), cations (+) and anions (-). The interaction between these phases results in the mechano-electrochemical phenomena required for cartilage maintenance (further information about the model formulation may be found in [7, 12, 13, 32]). A summary of the main model formulation is presented below.

#### Governing equations of the computational model of cartilage behaviour

It is important to note that the model has been validated and established in previous works [12, 13, 14]. Considering the four basic unknowns (**u**^s^ the displacement of the solid matrix, *ε*^*w*^ the chemical potential of water, *ε*^+^ and *ε*^*−*^ the electro-chemical potential for cations and anions, respectively), the four constitutive equations were established as:

*Momentum balance equation of the mixture*
  $$\nabla \cdot \underset{\sigma^{f}+\sigma^{c}+\sigma^{s}}{\underset{︸}{\sigma }}=0.$$ (3)
*Mass balance equation of the mixture*
  $$\nabla \cdot v^{s}+\nabla \cdot J^{w}=0.$$ (4)
*Charge balance equations of ions*
  $$\frac{\partial \left(\Phi^{w}c^{+}\right)}{\partial t}+\nabla \cdot \underset{\begin{array}{c} diffusion\\ of\;cations \end{array}}{\underset{︸}{J^{+}}}+\nabla \cdot \underset{\begin{array}{c} passive\;convection\\ \;of\;cations \end{array}}{\underset{︸}{\left(\Phi^{w}c^{+}v^{s}\right)}}=0,$$ (5)
  $$\frac{\partial \left(\Phi^{w}c^{-}\right)}{\partial t}+\nabla \cdot \underset{\begin{array}{c} diffusion\\ of\;anions \end{array}}{\underset{︸}{J^{{}^{-}}}}+\nabla \cdot \underset{\begin{array}{c} passive\;convection\\ of\;anions \end{array}}{\underset{︸}{\left(\Phi^{w}c^{-}v^{s}\right)}}=0,$$ (6)

In these equations, **σ** refers to the total mixture stress tensor whereas **σ**^*f*^, **σ**^c^ and **σ**^*s*^ are the stress tensors related to the fluid, chemical and solid phases, respectively. The velocity of the fluid matrix is defined as $v^{s}\;=\;\frac{\partial u^{s}}{\partial t}$. Regarding the ion concentrations, *c*^+^ and *c*^−^ denote cation and anion concentrations, respectively. Finally, *Φ*^*w*^ represents the porosity of the tissue [12, 13, 14]. The water flux, **J**^*w*^, cation flux, **J**^**+**^, and anion flux, **J**^**−**^, were expressed as a combination of the electrochemical potentials:

$$J^{w}=-\frac{R\;T\Phi^{w}}{\alpha }\left(\nabla \varepsilon^{w}+\frac{c^{+}}{\varepsilon^{+}}\nabla \varepsilon^{+}+\frac{c^{-}}{\varepsilon^{-}}\nabla \varepsilon^{-}\right),$$ (7)

$$J^{+}=-\frac{R\;T\Phi^{w}c^{+}}{\alpha }\nabla \varepsilon^{w}-\left[\frac{\Phi^{w}c^{+}D^{+}}{\varepsilon^{+}}+\frac{R\;T\Phi^{w}{\left(c^{+}\right)}^{2}}{\alpha \varepsilon^{+}}\right]\nabla \varepsilon^{+}-\frac{R\;T\Phi^{w}c^{+}c^{-}}{\alpha \varepsilon^{+}}\nabla \varepsilon^{-},$$ (8)

$$J^{-}=-\frac{R\;T\Phi^{w}c^{-}}{\alpha }\nabla \varepsilon^{w}-\left[\frac{\Phi^{w}c^{-}D^{-}}{\varepsilon^{-}}+\frac{R\;T\Phi^{w}{\left(c^{-}\right)}^{2}}{\alpha \varepsilon^{-}}\right]\nabla \varepsilon^{-}-\frac{R\;T\Phi^{w}c^{+}c^{-}}{\alpha \varepsilon^{-}}\nabla \varepsilon^{+},$$ (9)

Here, *α* refers to the drag coefficient between the solid and the water phases. The cation and anion diffusivities are represented by *D*^+^ and *D*^−^, respectively. The experimental atmospheric conditions were included in the model by *R*, the universal gas constant, and the absolute temperature, *T* [7]. Note that **J**^*W*^ refers to advection of the species with the moving pore fluid. However, **J**^+/−^ are the diffusion within and advection with the fluid flowing relative to the solid matrix.

The state variables appearing in Eqs 3–6 in terms of the basic variables of the problem may be also written as follows:

$$\sigma =\underset{\underset{osmotic\;pressure}{\underset{︸}{\sigma^{f}}}}{\underset{︸}{-PI}}\underset{\underset{GAGs\;repulsion}{\underset{︸}{\sigma^{c}}}}{\underset{︸}{-T_{c}I}}\underset{\underset{elastic\;stress}{\underset{︸}{\sigma^{s}}}}{\underset{︸}{+\lambda_{s}\theta I+2\mu_{s}\epsilon }},$$ (10)

$$\varepsilon^{w}=\frac{P}{R\;T}-\Phi \left(c^{+}+c^{-}\right)+\frac{B_{w}}{R\;T}\theta ,$$ (11)

$$\varepsilon^{+}=\gamma^{+}c^{+}e^{\frac{F_{c}\psi }{R\;T}},$$ (12)

$$\varepsilon^{-}=\gamma^{-}c^{-}e^{-\frac{F_{c}\psi }{R\;T}},$$ (13)

Where *F*_*c*_ is the Faraday constant, *Ψ* the electrical potential, *B*_*w*_ the fluid-solid coupling coefficient, *Φ* the osmotic coefficient, *γ*^+^ and *γ*^−^ the activity coefficient of the anions and cations, respectively and **I** the identity tensor. *P* is the fluid pressure, *θ* = div[**u**^**s**^] the solid matrix dilatation relating to the infinitesimal strain tensor of the solid matrix, *λ*_*S*_ and μ_*S*_ the Lame constants, and **ϵ** the infinitesimal strain tensor of the solid matrix [12, 13]. To consider the chemical expansion, *T*_*C*_, a new term was included in the mathematical formulation. This expansion was due to the presence of charge-to-charge electrostatic repulsive forces exerted on the GAG-collagen network (solid phase). Note that the equilibrium of cartilage swelling depends on the combined action of the chemical expansion stress, **σ**^*C*^, and the osmotic pressure. Both are related to the internal concentration and distribution of ions and their interaction with each other. Thus, *T*_*C*_ is represented as follows:

$$T_{c}=a_{o}c^{F}\text{exp}\left(-k\left(\frac{\gamma^{\pm }}{\gamma^{\pm^{*}}}\right)\sqrt{c\left(c+c^{F}\right)}\right),$$ (14)

Where *a*_*0*_ and *k* are the PG repulsion coefficients, and *c*^*F*^ the fixed charge density attached to PGs. *γ*^*±*^ and *γ*^±^* are the mean activity coefficients of ions during the process and in the reference state, respectively. *c* refers to the neutral external salt concentration [7].

#### Discretization

The tissue response in confined condition was solved using the finite element method. The primary unknowns of the model [**u**,*ε*^*w*^,*ε*^*+*^,*ε*^*−*^] were interpolated from nodal values. The time derivatives were approximated with the Crank-Nicolson method which yields an implicit approximation to the solution of the initial value problem *y*’ = *f*(*x*,*y*) with *y*(*x*_0_) = *y*_0_ at *x* for a given time step *h* [29]. To obtain the fully coupled non-linear system of equations describing the discretized model, we first established the weak formulation of the governing equations:

$$\int \nabla \delta_{u}\cdot \sigma \;dV=\int_{\Gamma }\delta_{u}\sigma^{*}n\;d\Gamma ,$$ (15)

$$\int \nabla \delta_{\varepsilon^{w}}\cdot v^{s}\;dV+\int \nabla \delta_{\varepsilon^{w}}\cdot J^{w}\;dV=-\int_{\Gamma }\delta_{\varepsilon^{w}}J^{w^{*}}\;n\;d\Gamma ,$$ (16)

$$\int \nabla \delta_{\varepsilon^{+}}\cdot J^{+}\;dV-\int_{Ω}\nabla \;\delta_{\varepsilon^{+}}\cdot J^{-}\;dV=\int_{\Gamma }\delta_{\varepsilon^{+}}\left(J^{{-}^{*}}-J^{{+}^{*}}\right)n\;d\Gamma ,$$ (17)

$$\int \delta_{\varepsilon^{-}}\frac{\partial \left(\phi^{w}c^{k}\right)}{\partial t}\;dV+\int \nabla \delta_{\varepsilon^{-}}\cdot J^{+}\;dV+\int \nabla \delta_{\varepsilon^{-}}\cdot J^{-}\;dV+\int \nabla \delta_{\varepsilon^{-}}\cdot \left(\phi^{w}c^{k}v^{s}\right)dV=-\int_{\Gamma }\delta_{\varepsilon^{-}}(J^{{+}^{*}}+J^{{-}^{*}})n\;d\Gamma ,$$ (18)

where **n** is the unit vector normal to the boundary and *δ*_**u**_, δ_**ε**_ $\delta_{\varepsilon^{w}},\;\delta_{\varepsilon^{+}}$ and ${\;\delta }_{\varepsilon^{-\;}}$ are the so-called test functions. The superscript * stands for the quantities in the bathing solution and *c*^*k*^ = *c*^+^ + *c*^−^ Eqs 14–17 were expressed in terms of primary variables in our previous works related to this model formulation and validation [12, 13, 14]

#### Numerical implementation

**Fig 3:**
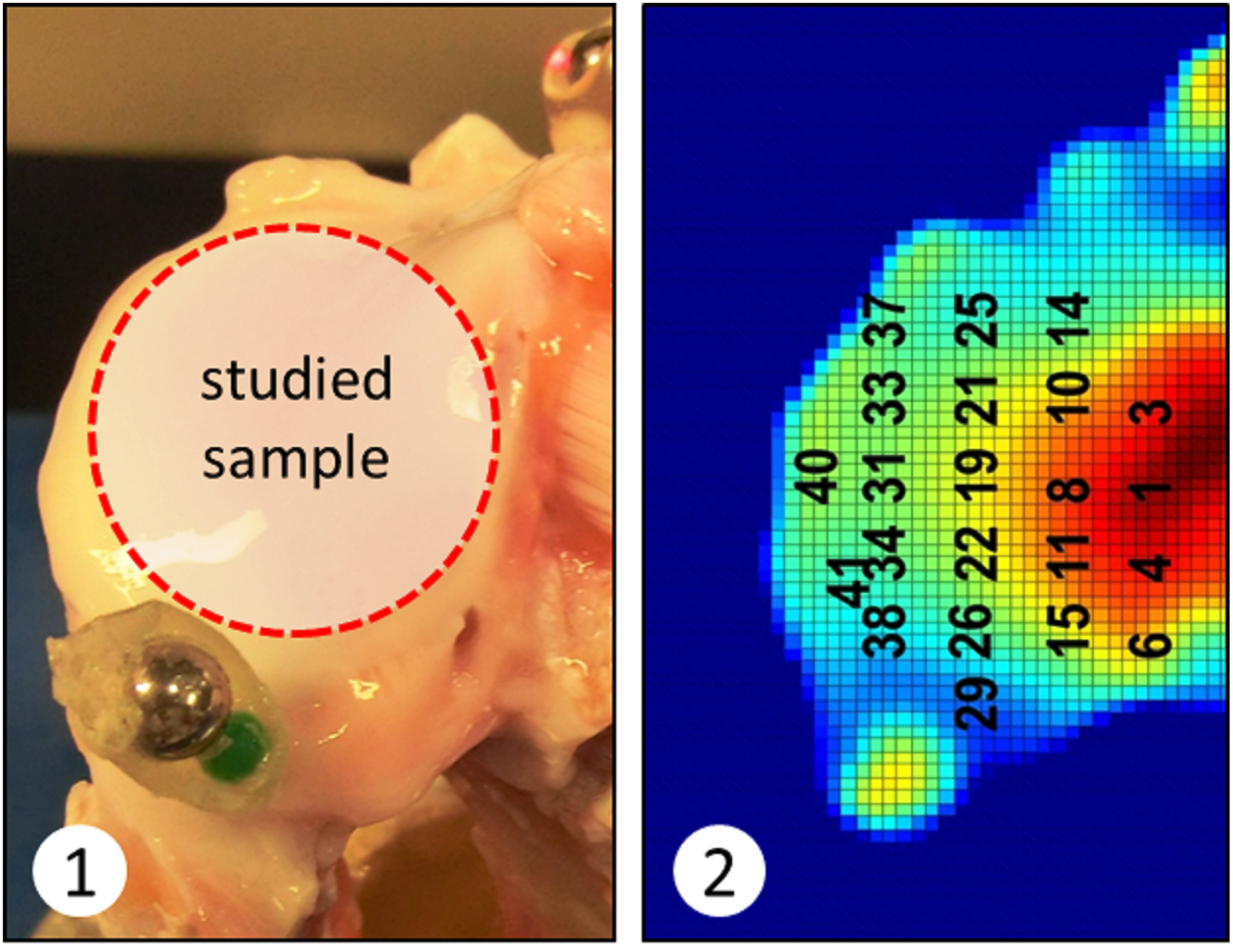
Schematic representation of the transient free swelling experiment (bottom right) and the boundary conditions of the cartilage sample applied to the computational simulation. A cartilage sample of 3 mm thickness and 30 mm diameter is immersed in a NaCl solution with an initial concentration of 0.15 M. The sample is confined in an impermeable chamber.

Tri-linear 8-noded hexahedral elements with 2×2×2 Gaussian integration points were used. The selected mesh resulted in 1680 elements. The finite element formulation described above was implemented in a user-defined element subroutine of the commercial software package Abaqus 6.11 (Dassault Systemes, Paris). To study the effect of through-the-thickness heterogeneity in cartilage behaviour, the experimental design described by Lai et al. [25] was computationally reproduced (Fig 3). Thus, a cartilage specimen of 30 mm diameter and 3 mm depth placed inside a circular confining ring was considered. The concentration of the external bath solution was decreased from 0.15 M to 0.125 M to mimic cartilage swelling observed in physiological conditions. No loads were applied on the sample in the z-direction. The properties of healthy homogeneous isotropic cartilage are listed in Table 1 while the properties of the through-the-thickness heterogeneous cases are listed in Tables 2 and 3. A comparison between the simulated results of the homogenous and the heterogeneous cases were performed. Quantification of cation fluxes and distributions within the samples and monitoring the tissue changes during the swelling processes were also achieved. Note that the homogeneous model $c_{0}^{F}$ was extracted from the literature [28] since this value was directly measured in the same kind of samples (porcine tibial plateaus) through a specific experimental procedure that provides the measure of the samples considering them as homogeneous as a whole. In this manner, side errors related to perform a mathematical approximation (medium of the measures of $c_{0}^{F}$ in layers) were avoided.

**Table 1:** 3D Model parameters for healthy cartilage considered as homogeneous material.

Description	Symbol	Value	Refs
Initial thickness of the sample	*h*_*sam*_	3.0 mm	measured
Initial diameter of the sample	*ϕ*_*sam*_	30 mm	measured
Young’s modulus	*E*	4.9·10^5^ Pa	measured
Poisson coefficient	*ν*	0.3	[29]
Drag coefficient between the solid and the water phase	*α*	7·10^14^ N·s·m^-4^	[28]
Diffusivity of the cations	*D*^*+*^	5·10^−10^ m·s^-1^	[28]
Diffusivity of the anions	*D*^*-*^	8·10^−10^ m·s^-1^	[28]
Initial FCD	$c_{0}^{F}$	0.222 mEq·ml^-1^	[28]
Activity coefficient of cations	*γ*^+^	1.0	[28]
Activity coefficient of anions	*γ*^−^	1.0	[28]
Gas constant	*R*	8.314 J·mol^-1^·K^-1^	[28]
Absolute temperature	*T*	298 K	[28]
Osmotic coefficient	*Φ*	1.0	[29]
Initial amount of water in the tissue	$\Phi_{0}^{w}$	0.8	[26]

**Table 2:** Initial permeability (*k*) introduced in the 3D computational model for healthy cartilage considered as through-the-thickness heterogeneous material.

Layer	Permeability (*k*) $\left(\frac{m^{4}}{N\cdot s}\right)$	Poisson coefficient, *ν*	Refs
Layer 1	1.21·10^−15^	0.12	[32]
Layer 2	1.14·10^−15^	0.34	[32]
Layer 3	1.07·10^−15^	0.45	[32]

**Table 3:** Initial fixed charged density ($c_{0}^{F}$) introduced in the 3D computational model for healthy cartilage considered as heterogeneous material based on the work of Lai et al. [25] as well as following our GAG-histological results. Property distribution similar to Young´s modulus represented in the zone of the sample shown in Fig 5. To clarify Table 3–Fig 5 correspondence, note that, for instance, first value in the first line and first column of table 3, 0.150, corresponds to the superficial layer and the point 13 of Fig 5. Values collected in table 3 are obtained in discrete material regions.

Initial fixed charge density, $c_{o}^{F}$ (mEq/ml)			
**Layer 1**			
0.150	0.150	0.150	0.150
0.175	0.175	0.175	0.175
0.180	0.180	0.180	0.180
0.200	0.200	0.200	0.200
**Layer 2**			
0.175	0.175	0.175	0.175
0.180	0.180	0.180	0.180
0.200	0.200	0.200	0.200
0.207	0.207	0.207	0.207
**Layer 3**			
0.180	0.180	0.180	0.180
0.200	0.200	0.200	0.200
0.207	0.207	0.207	0.207
0.210	0.210	0.210	0.210

#### Initial conditions

Initially, the cartilage sample was equilibrated with a single salt (NaCl) solution with concentration c*. The initial conditions for the computational model were:

$$t\;=\;0:\;u\;=\;0;{\;\varepsilon }^{w}\;=\;\varepsilon^{w^{*}};\;\varepsilon^{+}\;=\;\varepsilon^{{+}^{*}};\;\varepsilon^{-}\;=\;\varepsilon^{{-}^{*}}.$$

In this study, the free-swelling state of tissue equilibrated with the bathing solution was chosen as the reference configuration for strain (time 0 seconds, undeformed configuration).

#### Boundary conditions

The boundary conditions applied in the confined conditions are summarized below (Fig 3):

Free surface:
  $$\sigma_{z}\;=\;0;\;\varepsilon^{w}\;=\;\varepsilon^{w^{*}};\;\varepsilon^{+}\;=\;\varepsilon^{{+}^{*}};\;\varepsilon^{-}\;=\;\varepsilon^{{-}^{*}}.$$
Lateral surface:
  $$u_{x}\;=\;u_{y}\;=\;0;\;J_{x,y}^{w}\;=\;J_{x,y}^{+}\;=\;J_{x,y}^{-}\;=\;0.$$
Lower surface:
  $$u\;=\;0;\;J_{z}^{w}\;=\;J_{z}^{+}\;=\;J_{z}^{-}\;=\;0.$$

At t = 0 seconds the concentration of the external solution, c*, was increased from 0.15 M to 0.125 M. The transient response of the solid displacement was solved by using the new 3D model and compared to simulation results where heterogeneity and anisotropy of the cartilage are neglected.

## Results and Discussion

**Fig 4:**
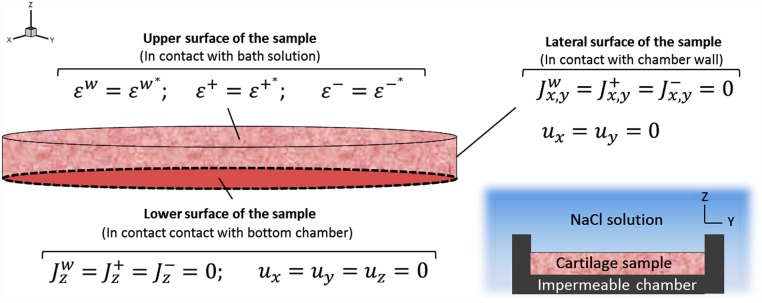
Schematic representation of the experimental tests carried out to obtain the Young´s modulus and GAG distribution (phase 1) for the 3D computational simulation (phase 2) of articular cartilage behaviour. Note that four random points were chosen to measure (qualitatively) the GAGs distribution.

The 3D computational model described above was used to simulate healthy articular cartilage behaviour in an external bath solution in confined conditions considering tissue through-the-thickness properties variation. First, an indentation test and histological analysis were performed on whole undamaged tibial plateau samples extracted from pigs to measure *E*_*s*_ along the cartilage surface. The GAG distribution was qualitatively and quantitatively assessed in the same locations as *E*_*s*_ Second, both parameters together with those extracted from the literature were introduced into the 3D mechano-electrochemical model to simulate tissue swelling. The results of the cartilage considered as heterogeneous were compared with those obtained in homogeneous and isotropic conditions (Fig 4).

From these results, we conclude that the use of computational models allows the swelling behaviour in cartilage to be studied in a non-invasive and inexpensive manner. In addition, the need to incorporate tissue inhomogeneities in the manufacturing process of cartilage-mimicking biomaterial can be guessed in line with the obtained results for cartilage where heterogeneity highly affects tissue simulations [30, 31, 33]. Similarly, this model provides healthy cartilage behavioural patterns that allow specific ranges of swelling to be established as a measure of tissue health.

Due to the lack of data for cartilage pig knee, $c_{0}^{F}$ in homogeneous case was extracted from literature where the same kind of sample were used and they are considered as homogeneous material in its whole. Thus, mathematical errors, derived from medium of values measured in layers, are avoided. On the other hand, $c_{0}^{F}$ in homogeneous case is slightly higher than maximum value of heterogeneous case, this suppose higher swelling of the sample. Importantly, differences between homogeneous and heterogeneous swelling, ion and water cases are observed providing essential information about how important is considering heterogeneity in cartilage simulation. Importantly, a model limitation could be found in the measurement of the mechanical properties of the samples superficial zone. This zone is considered to be solid matrix due to the preliminary exudation of water at the very beginning of the experiment. Other authors [34] offer different manner to perform this mechanical properties quantification. Similarly, it is important to remark that in the case of the homogeneous study, average fixed charged density has been extracted from literature, this can also affect in accuracy when comparing with heterogeneous case.

### Indentation tests

**Fig 5:**
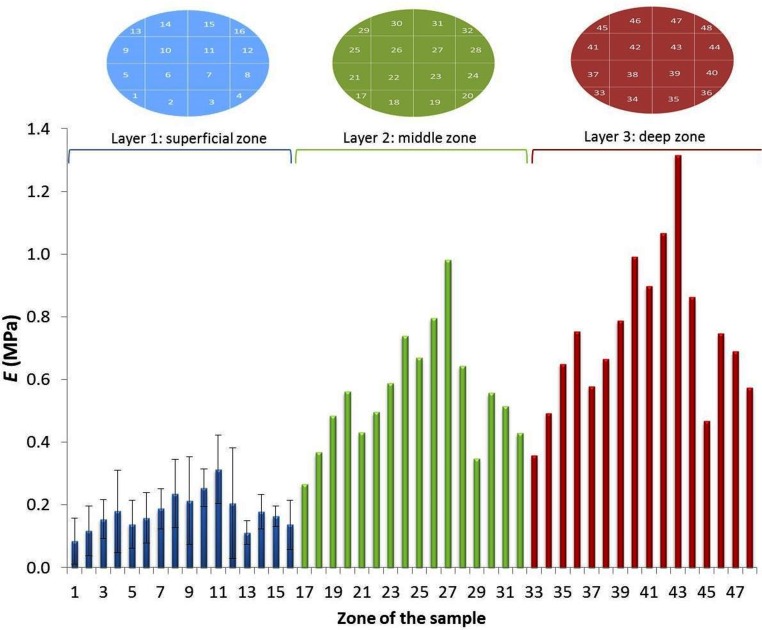
Young´s modulus experimentally obtained from the indentation test for the superficial layer (blue columns), and mathematically obtained for the middle zone (green columns) and deep zone (red columns) [19].

**Fig 6:**
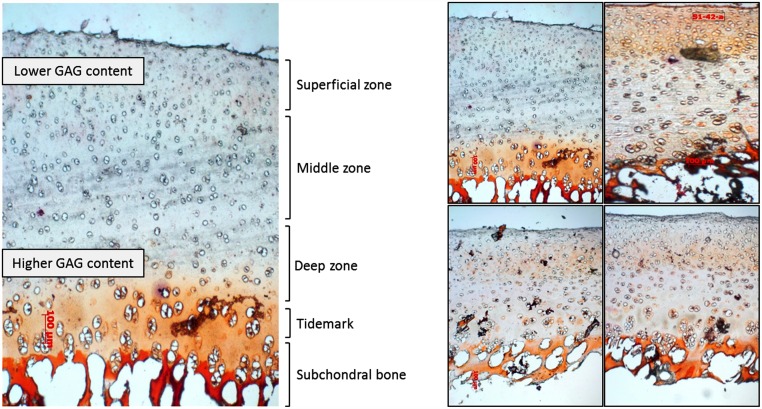
Panoramic view of a longitudinal section of the cartilage sample extracted from porcine tibial plateaus. Light microscopy of cartilage and subchondral bone, Safranin O staining, 100X.

The sixteen *E*_*s*_ measured on the whole cartilage surface are shown in Fig 5, where the blue columns correspond to the superficial zone. The resulting variation of *E* through the thickness was calculated by Eq 1 (Figs 5 and 6, green columns corresponding to the middle cartilage zone and red columns to the deep zone). Coincident with observations reported in the literature, zones located in the latero-posterior parts exhibited slightly lower values of *E* while this parameter was increased in deeper layers of the cartilage [29, 35, 36].

### Histological analysis

Histological sections stained with Safranin O were observed by light microscopy. Five zones were clearly identified according to the red colour grading. In the superficial zone, the grey tone indicates low GAG content whereas in deeper areas a high GAG concentration was detected. These findings corroborate experimental observations reported in the literature [25]. The resulting approximate pattern of the GAG arrangement and the subsequent fixed charge distribution in the superficial, middle and deep layers of the articular cartilage are shown in Fig 6. Specific fixed charge values are listed in Table 3, obtained experimentally from the histological GAG distribution. Values collected in Table 3 correspond to discrete material regions.

### Heterogeneous cartilage free swelling

To mimic physiological cartilage free swelling, the external bath concentration was decreased from 0.15 M to 0.125 M. Under this condition, the 3D computational model exhibited three distinct phases which were also observed in our previous works: I) an initial sample shrinking; II) a massive entrance of water into the sample during the first 1650 seconds of the free swelling simulation and III) a saturation of the sample, reaching the equilibrium state.

#### Z-displacement

**Fig 7:**
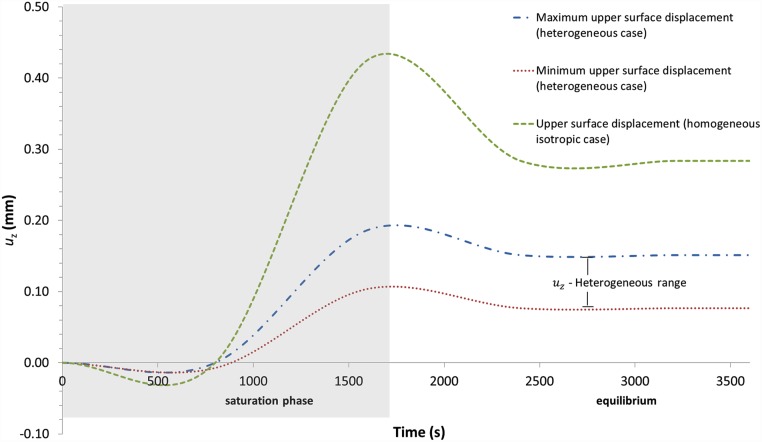
Comparison of the upper surface displacement in the free swelling test from the model considering articular cartilage as homogeneous material (green line) and through-the-thickness heterogeneous material (range marked by blue line, maximum value, and red line, minimum value). The dark area represents the saturation phase.

The physiological properties of healthy knee cartilage gave rise to a maximal displacement of 0.426 mm and 0.187 mm for the homogeneous and heterogeneous cases, respectively, within 1650 seconds of simulated time; subsequently in both simulations these values slightly decreased due to the minimum outgoing flux of cations, reaching a new equilibrium state at 2500 seconds at the end of simulation. This final state corresponds to 0.284 mm of z-displacement for the homogeneous case and 0.151 mm and 0.076 mm for maximal and minimal z-displacement, respectively, for the heterogeneous case (Fig 7). It is of importance to note in the latter case the substantially lower range of the z-displacement. Consistent with the behaviour of composite materials, when their properties have a heterogeneous distribution, the swelling of these materials is limited. Hence, irregular *E* and GAG distributions within the sample generate lower z-displacements. This finding has special relevance for biomaterials designed for the replacement of cartilage-damaged areas.

#### Cation distribution

**Fig 8:**
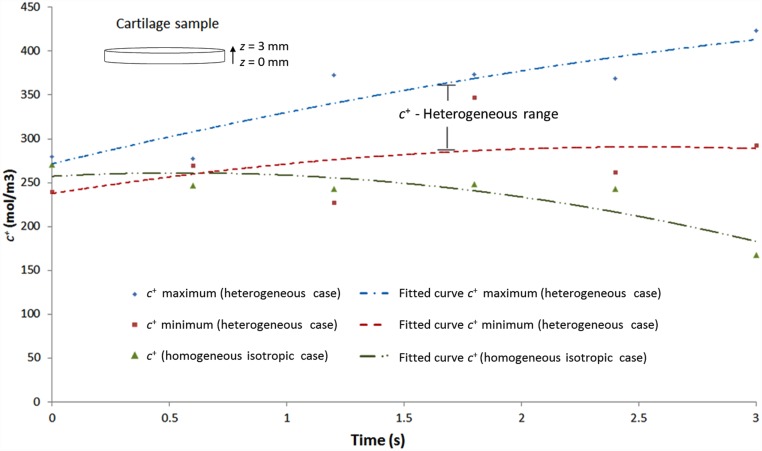
Range of cation concentration (*c*^+^) distribution in sample depth for the free swelling test considering cartilage through-the-thickness heterogeneity.

Under these conditions, the cation gradient was also monitored after 1650 seconds of free swelling, coincident with the maximum displacement time (Fig 8). The results are again compared with those obtained for the cartilage considered as homogeneous material. The latter case leads to a reduction in the cation concentration towards the surface of the sample, from 255 mol/m^3^ at *z* = 0 mm to 160 mol/m^3^ in the upper surface of the sample (*z* = 3 mm). In contrast, with varying through-the-thickness properties, this resulted in a wide range of cation distribution within the specimen, ranging from a maximum value of 410 mol/m^3^ in the upper surface to a minimum value of 245 mol/m^3^ at *z* = 0 mm. It is of importance to note that this alteration in the distribution of cations is due to the different resistance that cartilage offers to flow passage, which depends on the *E*_*s*_, GAG distribution and *k* values. This is an essential factor to consider when designing biomaterials for tissue repair, since it directly influences the distribution of nutrients and oxygen in the articular cartilage.

#### Fluxes and swelling

**Fig 9:**
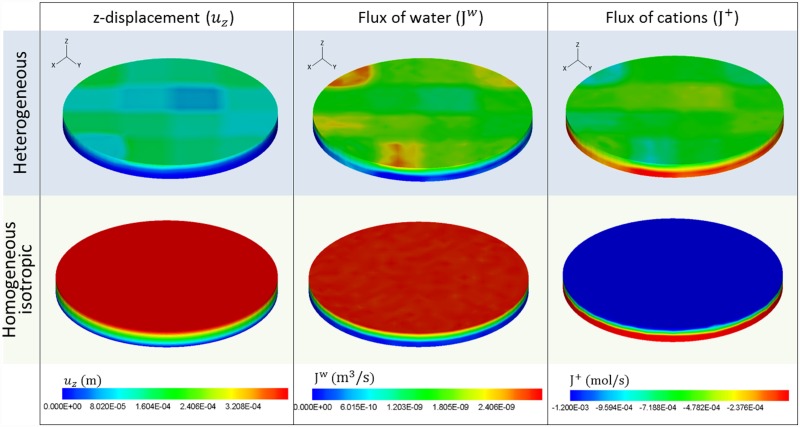
z-displacement of the upper surface of the cartilage, and the flux of water and cations obtained with the model after 1650 seconds (coincident with the maximum swelling of the sample) of the free swelling simulation for through-the-thickness heterogeneous materials and homogeneous materials. Note that the positive flux refers to the entrance of water or cations into the sample and the negative flux indicates the outflow of the substance.

The influence of anisotropy on cation fluxes and their distribution within the tissue was also considered. The simulation results are again shown again at 1650 seconds of the swelling for both numerical situations (through-the-thickness varying properties and homogeneous), corresponding to the main entrance of water (Fig 7). These results demonstrate that, similar to swelling, important variations in water and cation fluxes appear when considering anisotropy. In Fig 9, the heterogeneous results show how areas with lower *E*_*s*_ exhibit the highest entrance of water into the sample, 2.5·10^−9^ m^3^/s and subsequent higher z-displacements, whereas stiffer areas offer higher resistance to water entrance. Within the sample, the flux was reduced, the minimum being at the bottom of the sample (z = 0 mm). Biologically, this *E*_*s*_ variation within the cartilage is correlated with the collagen fibre distribution which enables resistance to higher shear stress. The GAG content influences the compression capacity of articular cartilage in the joint regions where direct cartilage-cartilage contact is found. Regarding the cation flux, the simulation shows how the cations flow out of the sample. Note that negative values indicate outflow. In the cartilage surface samples, the maximum and minimum outflows of cations were 9.57·10^−4^ mol/s and 2.37·10^−4^ mol/s, respectively. In Fig 9, the simulation results corresponding to the homogeneous case show homogeneous layers of water and cation fluxes. As expected, a regular water flux occurred in the cartilage, reaching its maximum value of 2.5·10^−9^ m^3^/s at the upper surface and decreasing towards the bottom of the sample. A maximum outgoing flux of 1.2·10^−3^ mol/s was observed in the upper surface. In both cases, anisotropy and isotropy water and cation fluxes were consistent with the external bath variation (from 0.15 m to 0.125 m). To equilibrate the imbalance between the internal sample medium and the external bath solution, an incoming flux of water and an outflow of cation fluxes was generated. These results show significant differences in water and cation fluxes when modelling cartilage as a heterogeneous material or as a simplified homogeneous material.

## Conclusions

This work presents an extension of a previously developed 3D mechano-electrochemical model used to analyse and quantify the effects of variations in through-the-thickness as well as radial properties on articular cartilage behaviour. The model is capable of predicting swelling and flow of water and cations as well as their distributions within samples when external bath solution varied. Furthermore, it enables us to quantify the influence of variations in the mechano-electrochemical properties on articular cartilage behaviour. This, in combination with its capability of displaying the results in easily interpretable 3D images, makes this model an interesting novel tool for designing and assessing the efficacy of biomaterials for cartilage repair. Additionally, it allows the establishment of baseline patterns from which tissue degradation due to disease can be assessed. The main novelty of the model is the incorporation of experimentally measured heterogeneous properties (*E*_*s*_ and GAGs distribution) which have been shown not only to vary in depth but also in the radial direction. In the absence of data in the literature regarding an accurate *E*_*s*_ distribution along the surface, we performed a systematic indentation test with the WASIM, an instrument specially designed to perform normal indentation on intact articular surfaces. A qualitative GAG distribution was obtained from histological assays of porcine specimens. The results show how the *E*_*s*_ as well as the GAG distribution increased in latero-posterior areas, coincident with zones that support higher compression and shear stress. These properties were introduced into the 3D computational model to determine their effects on the mechano-electrochemical events occurring in articular cartilage. The results obtained demonstrate that when considering cartilage as a heterogeneous material with variations in its through-the-thickness properties, the model predicts marked differences in swelling, fluxes and cation distribution compared to those obtained in homogeneous material simulations. There are significant reductions in the swelling and in the water and ion flux values compared to those previously obtained with samples having homogeneous properties. The computational results also showed that the upper surface displacement was twice as high in the isotropic case compared to the heterogeneous case where the stiffer areas offer higher resistance to material deformation. Similarly, the water and ion fluxes in the homogeneous case lead to higher flows that provoke the higher swelling. In the case of the cation distribution within the sample, the simulation evidenced how the heterogeneous case offers a wide range of cation distributions whereas in the homogeneous case, as expected, a homogeneous distribution within each layer was obtained. This indicates that fluxes are greatly affected by material heterogeneity and anisotropy providing areas with higher concentration of cations. In the light of the results obtained, mediated diffusive processes can be said to be highly influenced by the variation in properties generating zones with greater nutrient and oxygen up-take. It is therefore important to take into account through-the-thickness as well as radial variations in the material properties when manufacturing cartilage-mimicking biomaterials in order to achieve success in cartilage repair process.
